# Strain Diversity in the Human Microbiome: Personal Variation, Pathobionts, Therapeutics, and Methodological Challenges

**DOI:** 10.3390/microorganisms14030720

**Published:** 2026-03-23

**Authors:** Hyunjoon Park, Jung Soo Kim, Dong Joon Kim, Ki Tae Suk

**Affiliations:** 1 Institute for Liver and Digestive Diseases, Hallym University, Chuncheon 24252, Republic of Korea; hyunjoons.park@gmail.com (H.P.); jungsu052518@naver.com (J.S.K.); djkim@hallym.ac.kr (D.J.K.); 2 Department of Internal Medicine, Hallym Medical Center, Hallym University, Chuncheon 24252, Republic of Korea

**Keywords:** human microbiome, strain diversity, personalization, pathobionts

## Abstract

Advances in sequencing technologies have transformed human microbiome research, yet most analyses still rely on species-level profiles. However, strains rather than species represent the true ecological and functional units of the microbiome. Individual strains can vary substantially in gene content, metabolic capacity, virulence factors, antimicrobial resistance, and host-interaction properties. These differences critically influence immune responses, epithelial barrier integrity, disease susceptibility, and therapeutic outcomes. Here, we synthesize recent human microbiome studies that provide robust strain-resolved evidence, focusing on three major themes: (i) the emergence and long-term persistence of personalized strain repertoires, (ii) strain-specific pathobiont traits that drive host pathology, and (iii) the implications of strain-level ecology for the development of next-generation microbiome therapeutics. We also highlight key methodological innovations including high-resolution amplicon profiling, advanced metagenomic and single-cell genomics, and culture-based functional approaches that collectively enable strain-level resolution and are reshaping the field.

## 1. Introduction

Over the past decade, advances in sequencing, data informatics, and population-scale cohort analyses have fundamentally transformed our understanding of the human microbiome [1,2]. Shifting from early 16S rRNA amplicon sequencing, focused on taxonomic composition, to shotgun metagenomics, which unveiled the functional potential of microbial communities. This progression has expanded into integrative multi-omics approaches to capture actual microbial activities, and, most recently, these approaches are advancing toward precise mechanistic interactions between the host and specific microbial strains. These technologies have revealed that the conventional taxonomic units used to characterize microbial communities (from species to phylum) capture only a limited portion of the underlying biological complexity. A growing body of evidence now demonstrates that the most functionally consequential variation for host physiology, immune modulation, and disease susceptibility arises within species, at the level of genetically distinct microbial strains [3,4].

Microbial populations diversify through mutation, homologous recombination, horizontal gene transfer, and turnover of mobile genetic elements, giving rise to lineages with distinct metabolic capacities, environmental tolerances, virulence potentials, and host-interaction profiles [5]. In this review, we use the term “strain” to include both cultured isolates (“taxonomic strains”) and the genetically cohesive in situ populations delineated by metagenomic analyses (“natural strains”). Importantly, strains can exhibit profound differences in ecological behavior and functional consequences, often surpassing variation observed between species, yet these lineage-level distinctions remain largely obscured in conventional species-level microbiome profiling. A landmark example illustrating hidden within-species structure comes from the *Prevotella copri* complex described [6]. Although *P. copri* typically appears as a single species in 16S-based profiles, analysis of more than 6500 human gut metagenomes revealed four deeply divergent clades, each with inter-clade nucleotide divergence exceeding 10%, comparable to species boundaries. These clades, validated through isolate genomes, differ markedly in carbohydrate-active enzyme repertoires, indicating ecological niche partitioning within what is nominally a single species.

In this review, we synthesize recent human microbiome studies that provide robust, strain-resolved evidence. We first examine the ecological and evolutionary forces that generate and maintain personalized strain repertoires. Then highlight how pathogenic or pro-inflammatory traits map specific strains rather than entire species, emphasizing strain-defined pathobionts across major taxa. Next, we discuss how strain ecology shapes microbiome therapeutics, including fecal microbiota transplantation, defined microbial consortia, and phage therapy. Finally, we outline key methodological advances that enable strain-level resolution and are advancing microbiome research.

## 2. Personal Microbiome Variation

A central feature of strain-level microbiome organization is that individuals harbor deeply personalized collections of microbial lineages [7]. Understanding how these personal strain repertoires arise, persist, and change is essential for interpreting all downstream aspects of strain ecology from the emergence of pathogenic lineages to the predictability of therapeutic engraftment [8]. Personal variation reflects the ecological dynamics through which strains are maintained within individuals, exchanged among close contacts, and propagated across social networks [9].

### 2.1. Stability of Personal Strain Repertoires: Persistent but Not Immutable

Faith et al. [7] tracked oligotype-level variants using LEA-Seq and defined persistence as the repeated detection of the same sequence variant across longitudinal samples collected over multiple years, even when detection was intermittent. The results showed that 60–70% of strains detected at one time point persisted for years. Persistence was strongly stratified, with high-abundance lineages more consistently detected, whereas low-abundance strains were more transient. Genome sequencing of cultured isolates further demonstrated that homogeneous strains rarely occur among unrelated individuals but are enriched within families. Of course, the non-detection of low-abundance strains could be attributed to detection limits in both wet-lab and dry-lab methodologies. Importantly, however, these repertoires are not immutable. Sustained changes in host metabolic state, illness, antibiotic exposure, or prolonged cohabitation with other individuals can measurably reshape strain composition.

### 2.2. Origins of Personal Strains: Vertical Transmission

The early formation of the personal microbiome and the strains shared within families have been detailed in more depth. Ferretti et al. [10] employed shotgun metagenomics and strain-level tracking in 25 vaginally delivered mother–infant pairs, sampling multiple maternal body sites (gut, vagina, oral cavity, skin, and breast milk) in conjunction with the infant’s gut and oral microbiota during the first four months of life. Strain-level analysis reveals that identical strains are strongly enriched within mother–infant pairs compared with unrelated pairs, and that the maternal gut is the dominant donor for those lineages that persist across the sampling period during early infancy. Recent work further indicates that paternal microbiota also contributes to early-life strain acquisition. Dubois et al. [11] reported detectable strain sharing between fathers and infants consistent with paternal transmission and showed that paternal seeding can complement maternal transmission pathways, highlighting that early microbiome assembly reflects broader household microbial exchange rather than exclusively maternal inheritance. Vertical transmission, therefore, provides the initial scaffold of the personal strain repertoire.

### 2.3. Updating the Personal Microbiome: Household and Social Transmission

Personal strain repertoires are further reshaped across the lifespan through horizontal transmission. Valles-Colomer et al. extended the understanding of personal microbiomes to a global, population-scale view of strain-level transmission networks [9]. By integrating nearly ten thousand gut and oral metagenomes from 31 cohorts across 20 countries, they systematically quantified how often specific strains are shared between individuals with different types of relationships (mother–child, cohabitants, twins, community members, unrelated individuals). They showed that strain sharing between individuals is common and that patterns of strain similarity are structured by social relationships, consistent with microbial transmission and/or shared environmental exposure. The study addressed potential confounders by comparing related and unrelated individuals across multiple cohorts and geographic regions while applying strain-resolved genomic markers. The study also demonstrated that strain sharing persists well beyond infancy and is further shaped by horizontal transmission within households. Cohabiting adults shared substantially more gut and, even more markedly, oral strains than non-cohabiting individuals from the same population. Furthermore, twins who lived together in childhood retained overlapping strains decades later, whereas twins separated early showed reduced strain similarity. Strain overlaps declined progressively with social and geographic distance, indicating that personal strain repertoires are deeply socially structured.

### 2.4. Population Structure and Human Social Microbiome

Several studies have shown that the human gut microbiome has substantial within-species population structure. *Eubacterium rectale*, a dominant human gut butyrate producer, shows a clear global population structure with four geographically stratified subspecies, supporting a human-specific lineage that has co-dispersed with host populations [12]. Within-species population structure and local adaptation generate distinct ecological and functional profiles, thereby adding another layer of individuality to the personal gut microbiome beyond the species level. In parallel, the “social microbiome” concept provides a broader lens on how strain-level microbiota is assembled and maintained beyond individual hosts. Sarkar et al. [13] analyzed that hosts may be immunologically adapted to the shared, familiar microbiota circulating in their social group, which could reduce immune responses to those taxa. On the other hand, high similarity also means that once a pathogen (or a virulent strain of a pathobiont) manages to bypass colonization resistance and establish in one host, it is more likely to spread rapidly through the network because many individuals present comparable ecological niches for that pathogen. Thus, social convergence in microbiota composition has a double-edged role, simultaneously supporting mutual adaptation and increasing the risk of network-wide outbreaks when colonization resistance is breached.

**Figure 1:**
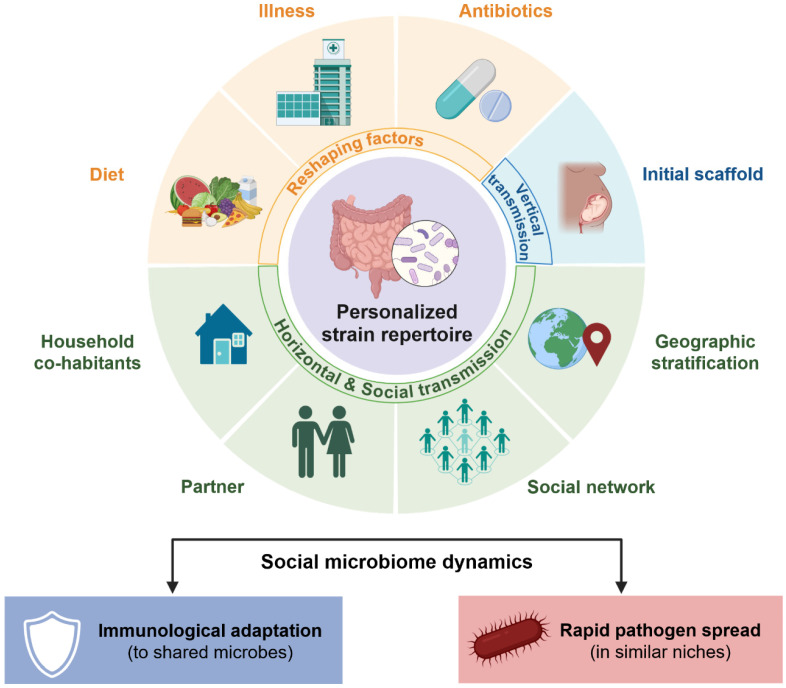
**Dynamics of personalized strain-level microbiome variation.** Personalized microbial strain repertoires exhibit ecological dynamics shaped by vertical and horizontal transmission and environmental factors and are continually reshaped. This dynamic balance between stability and exchange, connecting to microbiome-associated traits, can be patterned along family, social, and geographic lines. Created with BioRender.com.

Taken together, a population microbiome is structured by vertical seeding in early life and by household- and community-level interactions. Individual human hosts accept strain repertoires that are continually updated by social contacts, environmental exposures, and medical interventions. The persistence and turnover of strains within individuals are also shaped by ecological mechanisms, including niche partitioning, priority effects during early colonization, metabolic cross-feeding among community members, and competitive exclusion between closely related lineages. This dynamic balance between stability and exchange may help explain why microbiome-associated traits, such as disease susceptibility, vaccine responsiveness, and drug metabolism, can be patterned along family, social, and geographic lines (Figure 1).

## 3. Pathobionts

**Figure 2:**
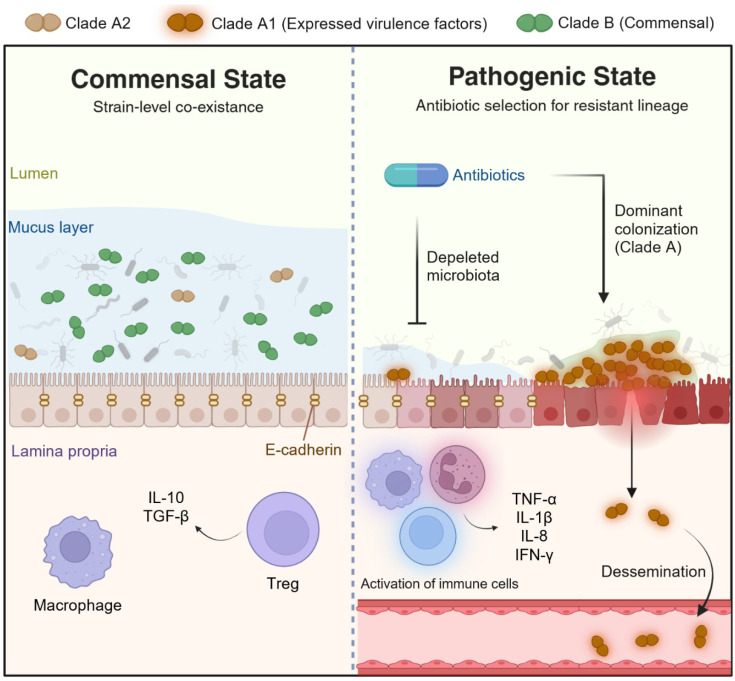
**Concept of pathobiont reaction modes in the human intestinal tract.** The *Enterococcus faecium* case shows that a species is a dynamic entity that can shift from commensal to pathogenic strain depending on its interactions with the host and environment. External triggers can also alter the pathogenic expression of pathobionts, inducing a transition to a pathogenic state. Created with BioRender.com.

Pathobionts typically inhabit the microbiota as commensals or opportunists yet possess lineage-specific genetic and functional traits that enable them to drive disease under specific ecological or host conditions (Figure 2). In this review, we adopt the widely accepted definition of a pathobiont as “a symbiont that is able to promote pathology only when specific genetic or environmental conditions are altered in the host [14].” Importantly, the pathobiont concept is inherently strain-dependent, as virulence-associated traits are often unevenly distributed across strains within the same species. These “pathobionts” highlight that disease risk is rarely a property of an entire species and is concentrated within evolutionary lineages. Below, we review representative examples that illustrate how strain-specific virulence factors, metabolic capabilities, and ecological strategies reshape host health.

Specific *Escherichia coli* strains harbor a ~50 kb pks pathogenicity island that encodes colibactin. This genotoxic metabolite alkylates adenines in DNA, inducing double-strand breaks and interstrand cross-links [15,16]. Long-term gut colonization of *pks^+^ E. coli* contributes causally to tumorigenesis [17]. The *pks^+^ E. coli* strains are detected in about 20% of healthy individuals and up to 60% of those with familial polyposis or colorectal cancer [18]. Interestingly, the traditional probiotic *E. coli* Nissle 1917 is also a colibactin-producing strain [19]. Large-scale metagenomic analyses further demonstrate that *E. coli* exhibits extensive strain-level genomic diversity in the human gut, with strains from different individuals sharing, on average, only ~80% of their gene repertoires and forming multiple phylogenetically distinct clades [20]. This duality further underscores why risk and function are strain-level properties, a concept critical for the rational design of therapeutics.

Enterotoxigenic *Bacteroides fragilis* (ETBF) promotes colon tumorigenesis via a strain-specific *B. fragilis* toxin (BFT), fragilysin activity. The fragilysin is a zinc-dependent metalloprotease that cleaves E-cadherin, disrupting epithelial cell–cell junctions. BFT exposure induces robust epithelial STAT3 activation and a Th17 immune response, accompanied by epithelial NF-κB signaling [21]. This drives CXCL chemokine production that recruits CXCR2^+^ immature myeloid cells (polymorphonuclear myeloid-derived suppressor cells, PMN-MDSCs) to the distal colon, thereby promoting tumor growth [22].

*Ruminococcus gnavus* is a common gut commensal, but it is enriched in patients with Crohn’s disease. Henke et al. [23] showed that many strains produce a defined cell-wall polysaccharide, glucorhamnan, which potently induces TLR4-dependent TNF-α responses, thus providing a plausible molecular link to intestinal inflammation. A follow-up study from the same group demonstrated that *R. gnavus* strains segregate into encapsulated and non-encapsulated types, and that a polysaccharide capsule can physically shield glucorhamnan and other inflammatory cell-wall components, yielding a far more tolerogenic host response [24]. Nishijima et al. reported large-scale analyses further suggest that some disease–microbe associations may be influenced by differences in fecal microbial load, indicating that enrichment of specific taxa should be interpreted cautiously when absolute microbial abundance is not considered [25].

Strain-level research in *Klebsiella pneumoniae* shows that pathobiont traits arise from specific lineages rather than the entire species. In NAFLD, only a subset of high alcohol–producing *K. pneumoniae* strains can ferment dietary carbohydrates into sufficient endogenous ethanol to induce steatosis, oxidative stress, and inflammatory liver injury in mice [26]. In contrast, conspecific strains with low or intermediate alcohol output do not exhibit this phenotype under comparable conditions. Complementarily, work on oral-derived *Klebsiella* strains has shown that only specific lineages can ectopically colonize the gut and drive a strong Th1-skewed mucosal immune response, precipitating colitis in genetically or microbiologically susceptible hosts [27]. Th1-driving capacity defines disease risk far more precisely than species-level presence or abundance.

Comparative genomics of diverse *Enterococcus faecium* isolates has revealed a clear population structure, in which a hospital-adapted, multidrug-resistant lineage (clade A1) has emerged from animal-and community-associated ancestors (clade A2), distinct from the predominantly commensal human gut lineage (clade B) [28,29]. Clades A and B likely reflect an ancient ecological split associated with the divergence of human and animal host niches [29]. In contrast, the divergence of clade A1 is a much more recent event, coinciding with the onset of intensive antibiotic use. Hospital-adapted clade A1 strains are characterized by expanded genomes that are enriched in mobile genetic elements, carbohydrate transport and metabolism functions, and multiple acquired resistance determinants, along with elevated mutation rates that facilitate rapid adaptation. In parallel, hospital-adapted lineages deploy an expanded arsenal of colonization and virulence factors, including specialized PTS carbohydrate uptake systems, adhesins, and surface polysaccharides that support gut colonization, biofilm formation, and survival in the bloodstream [30]. These virulence programs create a highly resilient opportunist that can persist in the hospital environment, resist antimicrobial assault, and exploit compromised hosts.

Across these examples, a consistent principle emerges that virulence factors, proinflammatory metabolites, and multidrug resistance determinants are encoded within specific lineages rather than within entire species. Thus, the epidemiological and clinical “unit of risk” is the strain, not the species. Recognizing this distinction is essential for accurate diagnostics, infection control, risk stratification, and the rational design of targeted microbiome therapeutics.

## 4. Therapeutic Implications

Microbiome therapeutics increasingly rely on an accurate understanding of strain-level ecology. Engraftment after fecal microbiota transplantation (FMT), the efficacy of defined microbial consortia, and the performance of next-generation probiotics all hinge not on species identity but on the specific strain lineages involved. Strain-level differences determine which lineages can colonize new hosts, compete with resident microbiota, produce key metabolites, or confer protection against pathogens. Below, we highlight how strain-resolved analyses have reshaped core therapeutic concepts across FMT engraftment, rationally designed consortia, and the development of next-generation probiotic and phage therapy.

### 4.1. FMT and Strain-Level Determinants

Clinical interest in FMT has surged in recent years, driving its application beyond the treatment of recurrent *Clostridioides difficile* infection (rCDI) to a multitude of therapeutic indications. FMT is a representative microbiome therapeutic approach in which strain-level analysis is conducted thoughtfully. In a study of FMT for rCDI, Smillie et al. [8] employed shotgun metagenomics and the strain-tracking model StrainFinder to analyze the microbial lineages of both donors and recipients. They observed a distinct “all-or-nothing” pattern of engraftment within species. Partial engraftment of only a subset of donor strains was relatively rare. The authors found the pattern to be reproducible across multiple rCDI clinical trial cohorts and even in an FMT study for metabolic syndrome. Therefore, the study emphasizes that identifying which species can robustly engraft in a given host-specificity must precede strain selection.

Another study on FMT engraftment has placed greater emphasis on strain specificity [31]. Using isolates from seven donors and thirteen recipients, the authors cultured 1008 strains spanning 207 species, sequenced their whole genomes, and defined strain-specific sequence markers to track the fate of individual lineages in shotgun fecal metagenomes before and after FMT. They found that, after successful FMT, approximately 71% of donor strains stably engrafted and could be detected for up to five years. In contrast, approximately 80% of pre-existing recipient strains were eliminated, and around 10% of ecological niches were filled by strains from environmental or other sources. By summarizing into a single metric, proportional engraftment of donor strains (PEDS), they demonstrated that the 8-week PEDS value alone could reliably discriminate between FMT success and clinical relapse (precision 100%, recall 95%). Specific taxa, particularly Bacteroides spp., consistently emerged as high-engraftment candidates across recipients. This study indicates that a single FMT can induce a near-permanent, strain-level reconstitution of the gut microbiota, providing a rational basis for designing defined live biotherapeutic products (LBPs) built from culturable strains with proven long-term engraftment capacity.

Podlesny et al. [32] performed a meta-analysis of 14 FMT trials across five indications using metagenomic strain profiling (SameStr) to quantify how donor, recipient, and ecological factors shape strain engraftment after FMT. They showed that donor-derived strains engraft preferentially when recipients are highly dysbiotic and low-diversity, and when protocols include broad-spectrum antibiotics, bowel lavage, and repeated FMTs, whereas high recipient α-diversity and a more intact microbiota favor persistence of resident strains. Conspecific coexistence of donor and recipient lineages within the same species was infrequent in the analyzed FMT datasets. These observations suggest that competitive exclusion frequently occurs during donor–recipient strain replacement under FMT conditions, although the balance between exclusion and coexistence may vary across species, ecological niches, and host contexts. And that engraftment probabilities vary systematically by bacterial lifestyle (for example, strict anaerobic gut commensals engraft far better than oral, oxygen-tolerant, or spore-forming taxa). On this basis, the authors argue for a conceptual distinction between “broad ecosystem restoration” FMT strategies, which deliberately maximize donor engraftment in heavily preconditioned recipients (as in rCDI), and “precision, strain-targeted” approaches for complex diseases. Collectively, these studies suggest that FMT donor and recipient selection and management should be considered at the strain level.

### 4.2. Defined Microbial Consortia

Defined microbial consortia can reproduce the therapeutic effects when they incorporate functionally critical strains. A consortium with *β*-lactamase-producing *Bacteroides* and *Parabacteroides* strains, *Clostridium bolteae*, and *Blautia producta*, which collectively prevented the expansion of vancomycin-resistant *Enterococcus faecium* (VRE), and decolonized VRE to levels comparable to those achieved through fecal microbiota transplantation [33]. In the consortium, the *Blautia producta* BPSCSK strain was found to possess a unique gene operon encoding a nisin-like lantibiotic, which was identified as the principal factor responsible for the direct killing of VRE. The study further demonstrated that, in an in vivo mouse model, the VRE-inhibitory efficacy of the microbial consortium was abolished when the BPSCSK strain was replaced with either a lantibiotic-deficient *Blautia* strain or *Lactococcus lactis*, which secretes nisin [34]. These studies show that therapeutic function is not evenly distributed across species; a single strain within a species can act as the keystone determinant of treatment efficacy. Thus, strain-level functional profiling is indispensable for rational consortia engineering.

### 4.3. Strain Heterogeneity in Next-Generation Probiotics

*Akkermansia muciniphila* is a mucin-degrading, mucus-resident gut bacterium that has emerged as a key next-generation probiotic linking the intestinal barrier to host metabolic and immune homeostasis [35]. *A. muciniphila* represents an open pan-genome species that comprises several phylogroups (AmI–AmIV) and candidate subspecies (Amuc1–Amuc4, AmucU) [36,37]. Individual *A. muciniphila* strains differ in key functional traits, including mucin and human milk oligosaccharide utilization, vitamin B_12_ biosynthesis capacity, and short-chain fatty acid production profiles [38,39,40,41]. Consequently, colonization by distinct *A. muciniphila* strains can elicit divergent effects on gut barrier integrity, low-grade inflammation, and host metabolic phenotypes, indicating that the species “*A. muciniphila*” masks substantial strain-level heterogeneity in host responses [35,42,43]. This clear functional divergence is similarly observed in other key commensals being developed as NGPs, such as the major butyrate producer, *Faecalibacterium prausnitzii*. *F. prausnitzii* is one of the butyrate-producing bacteria in the human gut. By providing a significant energy source for colonic epithelial cells and modulating host signaling pathways, such as NF-κB inhibition, PPARγ activation, and IFN-γ suppression, it is widely regarded as protective against colorectal cancer and inflammatory bowel disease (IBD) [44,45,46,47]. Within phylogroups with average nucleotide identity (ANI) values above 97%, robust differences have not yet been consistently demonstrated [48,49]. However, emerging multi-omics data indicate that aromatic amino acid-derived metabolites, short-chain fatty acid profiles, and associations with host metabolomic signatures differ between strains and phylogroups, suggesting that *F. prausnitzii* comprises functionally distinct subtypes with divergent host-interaction profiles [50,51]. In addition, numerous cohort studies report reduced total *F. prausnitzii* abundance and decreased phylotype/phylogroup richness in patients with IBD (Crohn’s disease and ulcerative colitis), colorectal cancer, selected subtypes of irritable bowel syndrome, and metabolic disorders such as obesity and type 2 diabetes. The pattern of which phylogroup is most depleted varies according to disease entity and the anatomic location of inflammation [52,53]. For example, depletion of phylogroup I emerges as a common signature across several intestinal disorders. In contrast, a more pronounced reduction in phylogroup II has been particularly associated with Crohn’s disease involving the ileum and with a history of ileal resection [51,53,54]. Together, these examples highlight that NGP development requires strain-level rather than species-level inference, as within-species diversity can fundamentally alter therapeutic potential.

### 4.4. Strain Issues in Phage Therapy

**Figure 3:**
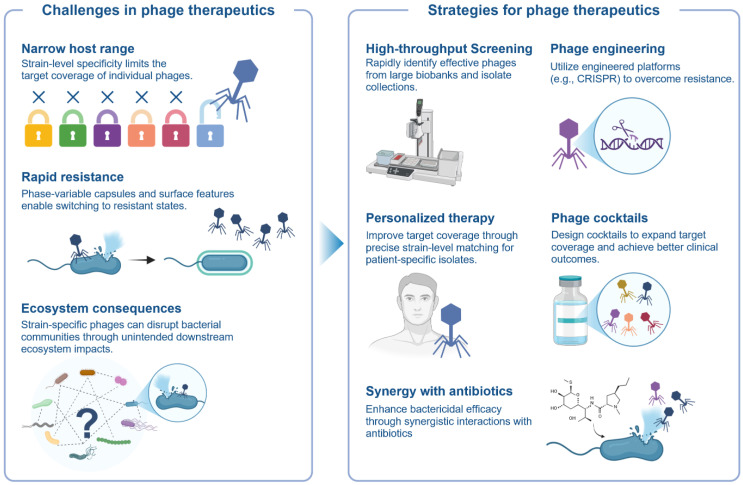
**Phage therapeutics and the bacterial strain-diversity challenge.** The inherent strain specificity of phages offers therapeutic precision, but it is accompanied by the significant challenge of considering clinical availability. Created with BioRender.com.

Phage therapeutics for microbiome modulation are fundamentally constrained and enabled by strain-level specificity (Table 1). Recent gut-focused resources, including large isolate collections and dedicated phage biobanks, systematically map infection matrices across many bacterial strains and provide the empirical basis for selecting or designing effective phage cocktails [55,56,57]. These studies consistently show that, even within a single bacterial species, susceptibility can vary markedly across strains, suggesting that strain-level matching and cocktail design could improve target coverage and efficacy [58,59,60]. Furthermore, phase-variable capsules and other surface features can switch between permissive and resistant states, complicating the effective bactericidal action of target bacteria by phage [59,60]. Mechanistic insights into these dynamics are informing next-generation strategies, including engineered phage platforms such as phage-delivered CRISPR systems [61,62]. Genetically engineered phage cocktails have shown improved therapeutic potential compared with conventional wild-type phage cocktails, although research and clinical translation remain at an early stage. Another consideration is that even highly specific phages can disrupt bacterial communities through direct and/or indirect interactions among bacteria, underscoring the need to evaluate both on-target efficacy and downstream ecosystem consequences [63]. In summary, phage therapeutics are being researched to meet comprehensive requirements, including strain coverage, bactericidal efficacy, stability, and impact on the microbiome (Figure 3).

## 5. Methodological Landscape

Strain-resolved microbiome analysis has emerged through the convergence of sequencing technologies, computational frameworks, and culture-based genomics. Conceptually, there is no single method that provides a complete view of strain-diversity. Instead, each platform captures a different facet of strain-associated variation, with distinct strengths and blind spots, and they are more powerful when combined (Table 2).

### 5.1. High-Resolution Amplicon Sequencing

High-resolution amplicon methods extend classical 16S rRNA profiling by explicitly modeling sequencing errors and sub-OTU structure. Minimum Entropy Decomposition (MED) and error-model-based denoising pipelines such as DADA2, Deblur, and UNOISE3 collapse noisy reads at single-nucleotide resolution [64,65,66,67]. These methods reveal within-species diversity that is invisible at conventional 97% identity of 16S amplicon-based OTU cutoffs. They are beneficial for longitudinal tracking of dominant lineages and for identifying fine-scale associations between strain-like variants and host phenotypes in large cohorts.

### 5.2. Shotgun Metagenomics

Shotgun metagenomic approaches provide richer genomic information and support several complementary modes of strain-level inference. Marker-based frameworks such as MetaPhlAn and StrainPhlAn reconstruct sample-specific consensus sequences for clade-specific markers and then infer phylogenetic relationships among dominant strains across samples [68,70]. Pangenome-based tools such as PanPhlAn instead model the presence or absence of thousands of gene families per species, yielding strain-level gene repertoires [20]. SNV-based deconvolution methods, including ConStrains, StrainFinder, StrainGE, inStrain and related frameworks, use single-nucleotide polymorphism patterns in conserved genes or across whole genomes to infer multiple conspecific haplotypes and their relative abundances within a sample [69,71]. These methods can disentangle mixtures of strains, track clonal replacements, and quantify transmission and persistence even when strains occur at low coverage. However, the inaccuracy and incompleteness of raw sequences of shotgun sequencing remain a problem.

### 5.3. Single-Strain Genomics

More recently, single-strain genomics has begun to close the gap between population-level MAGs and true single-strain genomes. Microfluidics-based platforms such as Microbe-seq encapsulate individual bacterial cells in droplets for lysis and whole-genome amplification (WGA), generating thousands of single-amplified genomes (SAGs) from a single stool sample [73]. Large SAG catalogs from oral and gut microbiomes have revealed extensive strain-level diversity and uncovered previously unrepresented taxa in shotgun MAG collections [74]. Hybrid frameworks such as SMAGLinker jointly leverage the specificity of single-cell data and the contiguity of bulk metagenomes [75]. These single-strain genomics provide a powerful culture-independent route but remain technically demanding and are still affected by WGA bias and incompleteness.

### 5.4. Culture-Dependent Genomics

Culture-dependent genomics provides high-quality and complete genomes. However, a critical challenge lies in efficiently acquiring hundreds to thousands of isolates to cover the vast diversity of the human microbiome. The high-throughput isolation and identification strategy, “culturomics,” systematically explores diverse media, atmospheric conditions, and physical treatments to recover thousands of gut isolates, which can then be identified by MALDI-TOF mass spectrometry or sequencing and subjected to whole-genome analysis [76]. Isolate-based genomics offers the highest confidence, and each strain can be subjected to functional assays, including high-throughput phenotyping, metabolomics, transposon mutagenesis, and gnotobiotic colonization experiments.

## 6. Conclusions

Evidence from longitudinal, familial, and population-scale studies demonstrates that the human microbiome is organized at the level of distinct microbial strains, which form personalized, yet socially shared repertoires shaped by vertical transmission, cohabitation, and ecological filtering. These personalized strain repertoires provide the ecological context in which specific microbial lineages function as either beneficial symbionts or pathobionts that can drive disease under host or environmental conditions. Across multiple domains—including pathobiont biology, fecal microbiota transplantation (FMT), microbial consortia, and emerging next-generation probiotics—strain-resolved analyses consistently demonstrate that disease risk, transmissibility, and therapeutic efficacy arise from specific microbial lineages rather than broad species-level classifications. These findings highlight the importance of strain-level resolution for understanding microbiome ecology and for designing effective diagnostics and interventions. Methodological advances, including high-resolution metagenomics, microdiversity computational tools, and large-scale culture-based microbiome resources, are increasingly making strain-level investigation feasible at the population scale. Integrating these approaches with longitudinal sampling and experimental validation will be essential for linking strain variation to functional and clinical outcomes.

Moving forward, a key priority for the field will be the systematic integration of strain-resolved multi-omics with culture-based experimentation and curated microbial isolate collections. Such efforts will enable the development of precision microbiome diagnostics, surveillance frameworks for pathobiont lineages, and rationally designed live biotherapeutic products.

**Figure 4:**
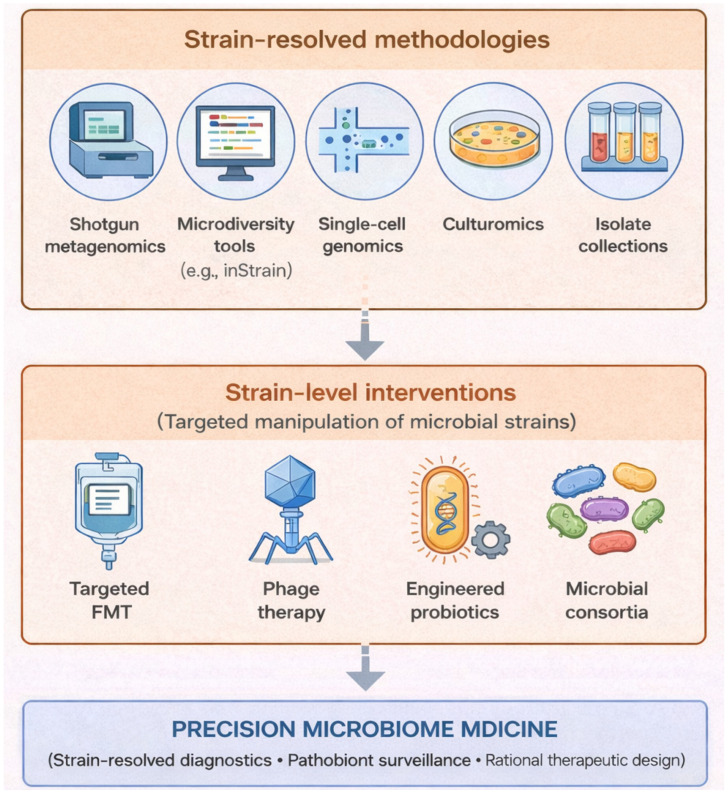
**Strain-resolved strategies enabling precision microbiome medicine.** Strain-level understanding of human microbiota improves the outcomes of microbiome therapeutics interventions such as fecal microbiota transplantation, phage therapy, and engineered microbial therapeutics. Advances in strain-resolved methodologies provide the foundation for understanding and manipulating microbiomes at the level of individual strains. Created with generative AI (ChatGPT 5.3).

Although this review focuses primarily on bacterial strains, strain-level variation is increasingly recognized in other microbiome components, including fungal strains, archaeal lineages, and viral variants, where intra-species diversity can similarly influence ecological behavior and host interactions (Figure 4).

## Figures and Tables

**Table 1:** Strain-level considerations for microbiome-targeted phage therapeutics.

Category	Target	Strain-Level Issue	Reference
Host/Strain Specificity & Phage Heterogeneity	Gut commensals	Susceptibility varies strongly across strains/species	[55]
	Carbapenem-resistant *K. pneumoniae* (gut resident)	Cocktail design depends on target species/strain collection scale	[56]
	Gut commensals	Host specificity + condition-dependence (e.g., oxic/anoxic infectivity)	[57]
	*Bacteroides thetaiotaomicron* (multiple strains)	Predicting host range requires strain-resolved data (especially CPS type)	[58]
	*B. thetaiotaomicron*	Phase-variable CPS/lipoproteins shift phage susceptibility	[59]
	*B. intestinalis* + crAss-like phage	CPS variation & pseudolysogeny within strain	[60]
Engineered Precision Targeting	*E. coli* + M13 (CRISPR/cas9)	Spacer deletion, target site mutation Very low efficiency, stability, coverage	[61]
	*E. coli* + cocktail (tail fiber engineering, CRISPR-armed multiple genes targeting)	Required bactericidal efficacy and broad clinical strain coverage	[62]
Ecological Consequences	Defined gut community in gnotobiotic mice	Removing one strain can cascade to others + metabolites	[63]

**Table 2:** Culture-independent methods and tools for strain-level or strain-associated human microbiome analysis.

Platform	Method	Concept and Feature	Limitations
Amplicon sequencing	MED [64]	Shannon entropy-based unsupervised oligotyping; very high resolution within OTUs; no pairwise distance matrix; scalable to large datasets; unsupervised	Strongly dependent on QC and parameters; Does not fully remove PCR/sequencing artifacts
	Deblur [65]	Greedy subtraction using Hamming distance; per-sample denoising; single nucleotide resolution OTU; enabling easy scaling and cross-run integration; good stability across technical replicates and sequencing rounds	Requires stringent upstream QC (quality trimming, chimera removal, fixed read length); like other denoising tools, cannot fully correct for PCR/chimera artifacts
	DADA2 [66]	Statistical error model learned from data; very accurate, model-based denoising; widely used and well-documented; rich downstream tools in R	Slower and more memory-intensive than Deblur/UNOISE; run-wide error learning can be heavy on huge projects; primarily for Illumina amplicons
	UNOISE2/3 [67]	Abundance-based denoising with simple error model; very fast and lightweight; single-nucleotide resolution; simple workflow	Original USEARCH implementation is not fully open for all use; less explicit modeling of errors/Q-scores
Shotgun metagenomics	PanPhlAn [20]	Pangenome-based profiling; useful for functional comparison of strains and epidemiology studies	Focuses mainly on the dominant strain; limited ability to deconvolve mixtures of multiple strains of the same species
	MetaMLST [68]	in silico MLST; assigns sequence types (STs) or novel STs based on allele combinations; Directly links metagenomes to classical MLST epidemiology	Generally reports only the dominant strain/ST; variation outside MLST loci is not captured
	ConStrains [69]	SNP frequency patterns in universal or species-specific genes; infer multiple conspecific strain haplotypes and their relative abundances within a sample; well-suited for FMT, longitudinal, or within-host dynamics studies	Requires high coverage; not suitable for rare species. Only a subset of the genome (marker genes) is modeled, so full gene content and functions are not directly inferred
	StrainPhlAn [70]	Reconstruction sample-specific consensus sequences of MetaPhlAn clade-specific markers; multiple-sequence alignment and phylogenetic analysis; Optimized for strain tracking, transmission, and large-scale strain phylogeny in big cohorts; integrates naturally with the bioBakery platform	Assumes a single dominant strain per sample and does not deconvolve mixtures; restricted to species represented in the MetaPhlAn marker catalog
	StrainGE [71]	Reference-based strain toolkit (StrainGST: identify reference genomes; StrainGR: call SNVs and large deletions); enabling nucleotide-level comparison and tracking of multiple conspecific strains, even at low coverage; Strong for deconvolving strain mixtures; characterizing SNVs/gaps	Reference-dependent: performance and resolution are limited by the quality and diversity of the reference genome database; less suitable for species with sparse or biased references
	inStrain [72]	Population microdiversity analysis using genetic metrics (nucleotide diversity, SNV density, allele frequency spectrum, linkage disequilibrium); high-stringency strain comparison	Reference-dependent; haplotype reconstruction limitation in short-read; sequencing depth dependency; not for complete strain genome
Single-strain genomics	Microbe-seq [73]	High-throughput microfluidic single-cell WGS; pooled sequencing yields many SAGs that can be co-assembled into strain-resolved genomes	SAGs can be incomplete and biased due to WGA; genome recovery rate per cell is still limited, and setup (microfluidics, WGA QC) is experimentally demanding
	bbsag20 [74]	Reference SAG catalog; Curated catalog of 17,202 medium-or-better SAGs (plus MAGs) from oral and gut microbiomes; maps mobilome–resistome networks to individual host strains	Not including the downstream tool, just a fixed dataset; focused on Japanese cohort and SAG-gel platform, still subject to SAG completeness/contamination constraints
	SMAGLinker [75]	Integration framework (SAG + metagenome); merges SAG- and metagenome-derived bins into high-quality, strain-resolved genomes; clarifying metabolic pathways and horizontal gene transfer networks	Requires both single-cell and metagenomic sequencing from the same samples; performance depends on SAG quality and coverage

## Data Availability

No new data were created or analyzed in this study. Data sharing is not applicable to this article.

## Abbreviations

The following abbreviations are used in this manuscript:

AMR	Antimicrobial Resistance
ANI	Average Nucleotide Identity
ASV	Amplicon Sequence Variant
BFT	Bacteroides fragilis Toxin
CPS	Capsular polysaccharides
CRC	Colorectal Cancer
CXCL	C-X-C motif chemokine ligand
CXCR2	C-X-C motif chemokine receptor 2
ETBF	Enterotoxigenic Bacteroides fragilis
FMT	Fecal Microbiota Transplantation
IBD	Inflammatory Bowel Disease
LBP	Live Biotherapeutic Product
LEA-Seq	Low-Error Amplicon Sequencing
MAG	Metagenome-Assembled Genome
MED	Minimum Entropy Decomposition
MLST	Multilocus Sequence Typing
NAFLD	Nonalcoholic Fatty Liver Disease
NGP	Next-Generation Probiotic
NTBF	Non-toxigenic Bacteroides fragilis
OTU	Operational Taxonomic Unit
PEDS	Proportional Engraftment of Donor Strains
PMN-MDSC	Polymorphonuclear Myeloid-Derived Suppressor Cell
rCDI	Recurrent Clostridioides difficile Infection
SAG	Single-Amplified Genome
SNP	Single-Nucleotide Polymorphism
SNV	Single-Nucleotide Variant
STAT3	Signal Transducer and Activator of Transcription 3
VRE	Vancomycin-Resistant Enterococcus
WGA	Whole-Genome Amplification
